# Supplementary figures and images for: Analysing the effectiveness of Twitter as an equitable community communication tool for international conferences
Source: PeerJ. 2023 May 8;11:e15270. doi: 10.7717/peerj.15270 (PMC10174057; doi:10.7717/peerj.15270)

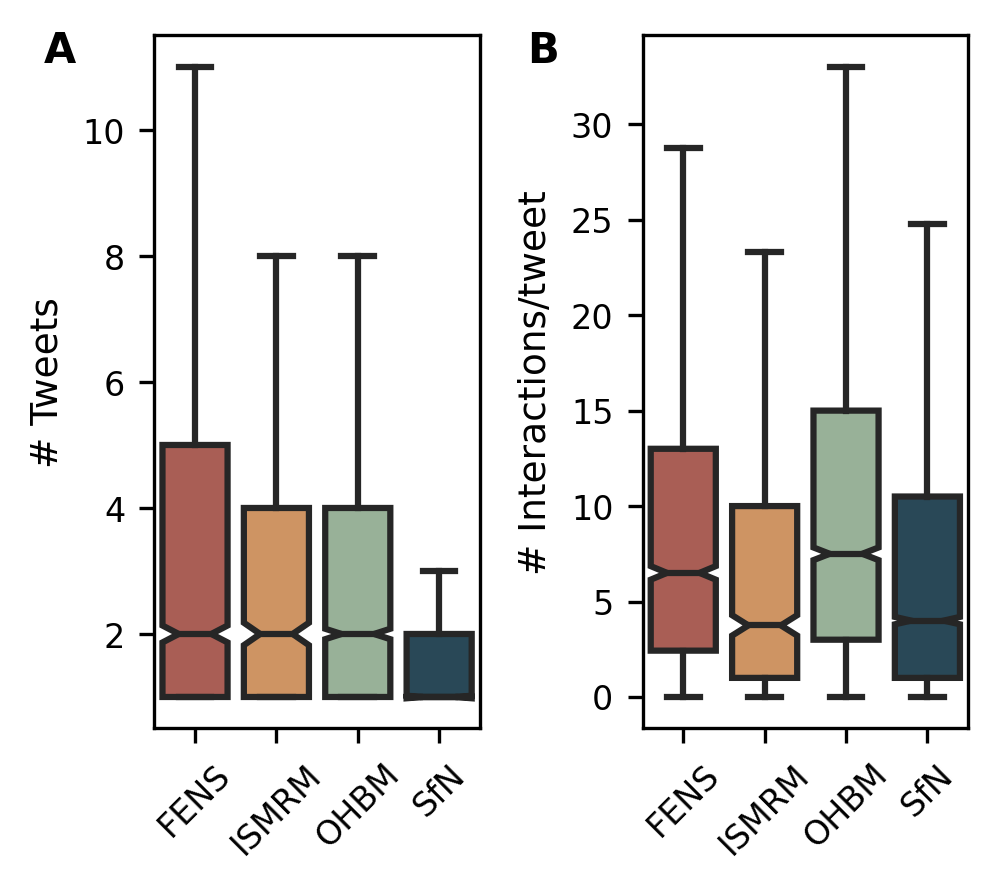

Supplement: Supplemental Information 1 — Number of (A) tweets sent and (B) interactions per tweet that each user had for each of the four conferences studied. [file peerj-11-15270-s001.png]

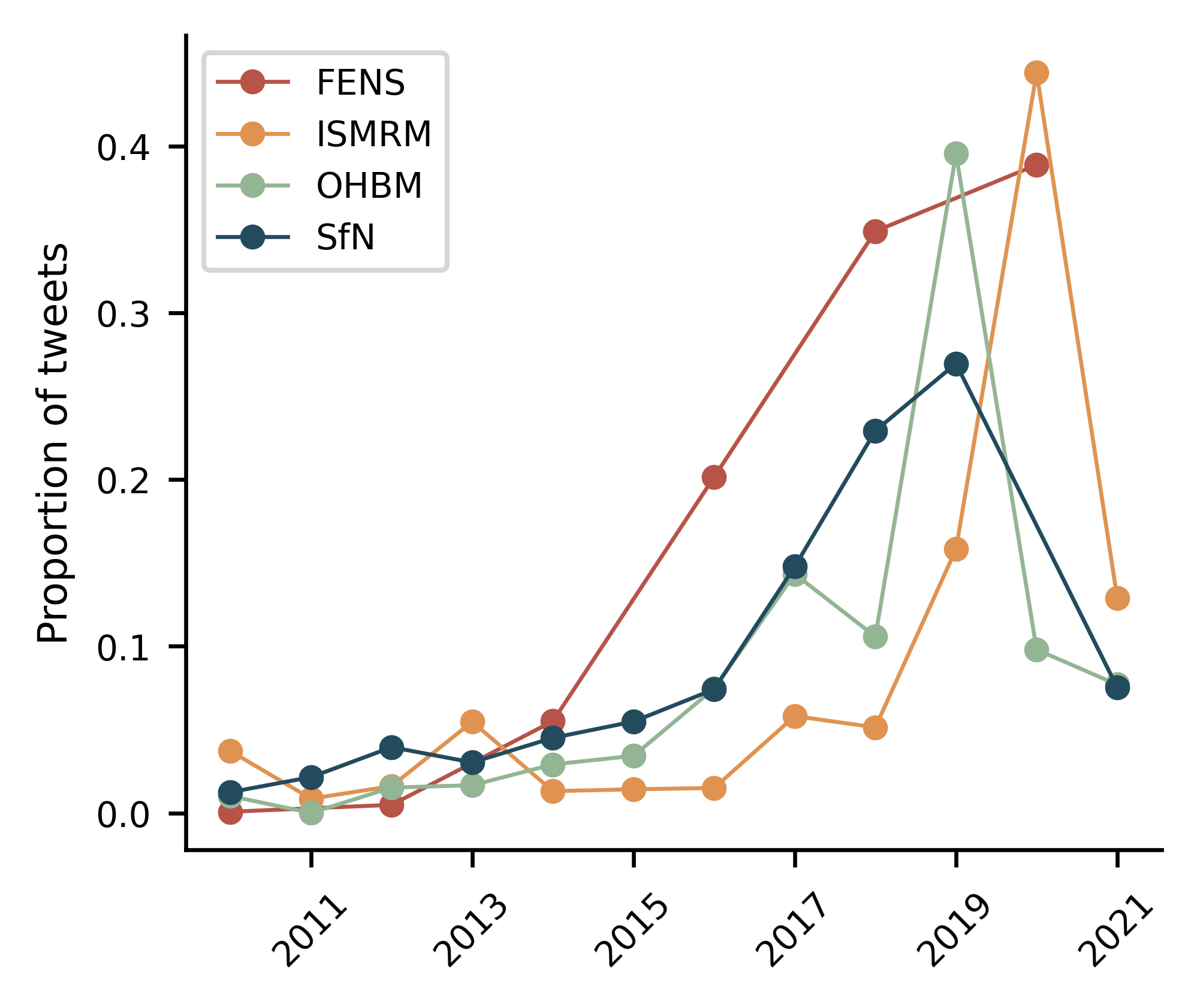

Supplement: Supplemental Information 2 — Number of tweets sent per year for each conference separately, normalised by the total number of tweets sent for that conference. [file peerj-11-15270-s002.png]

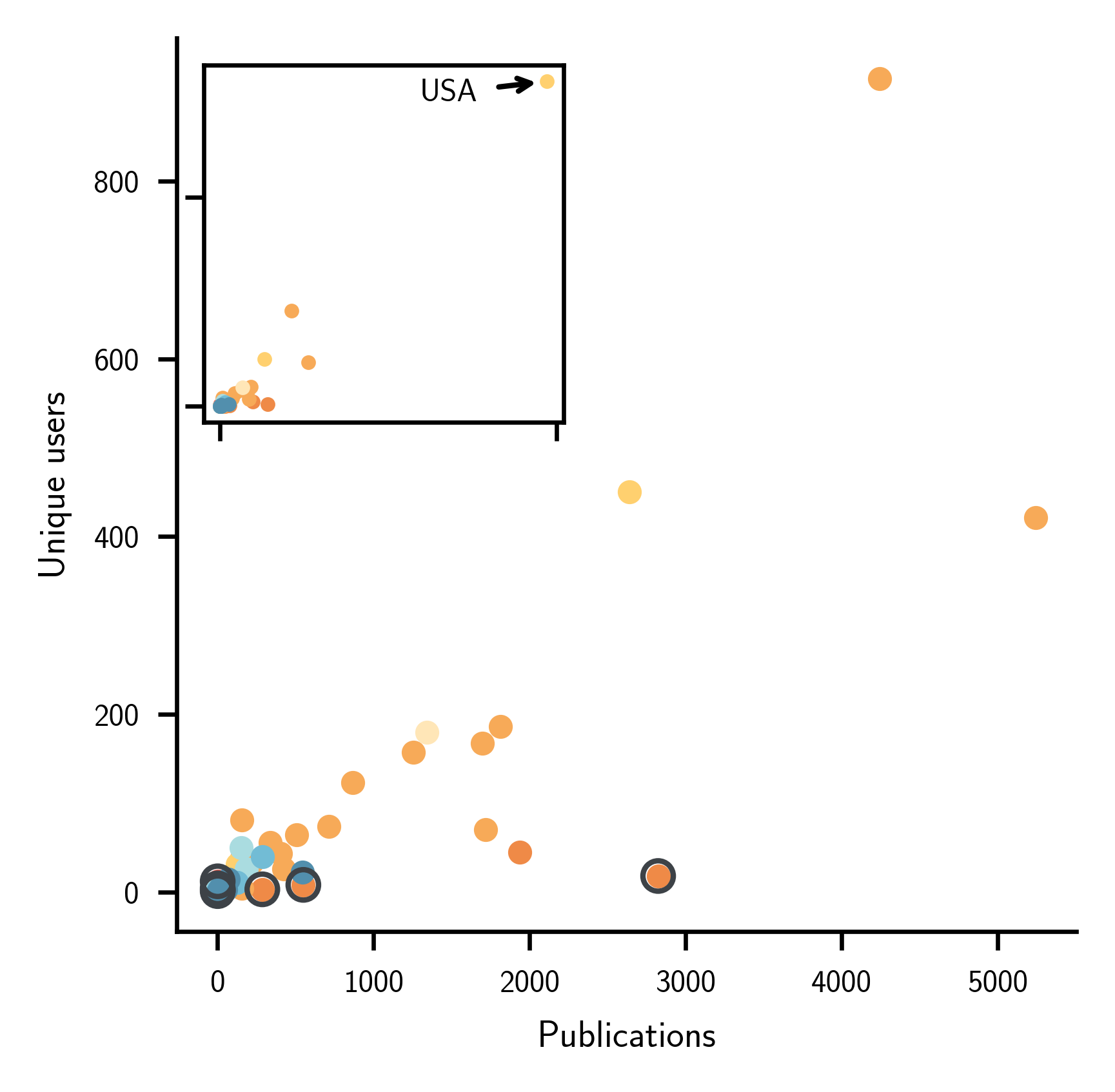

Supplement: Supplemental Information 3 — Publication and user numbers for each country plotted in original space. The main figure excludes the USA, with that country shown in the inset. Countries where the number of users is unexpected given the number of publications are highlighted in a dark circle. Countries are shaded according to their region. [file peerj-11-15270-s003.png]

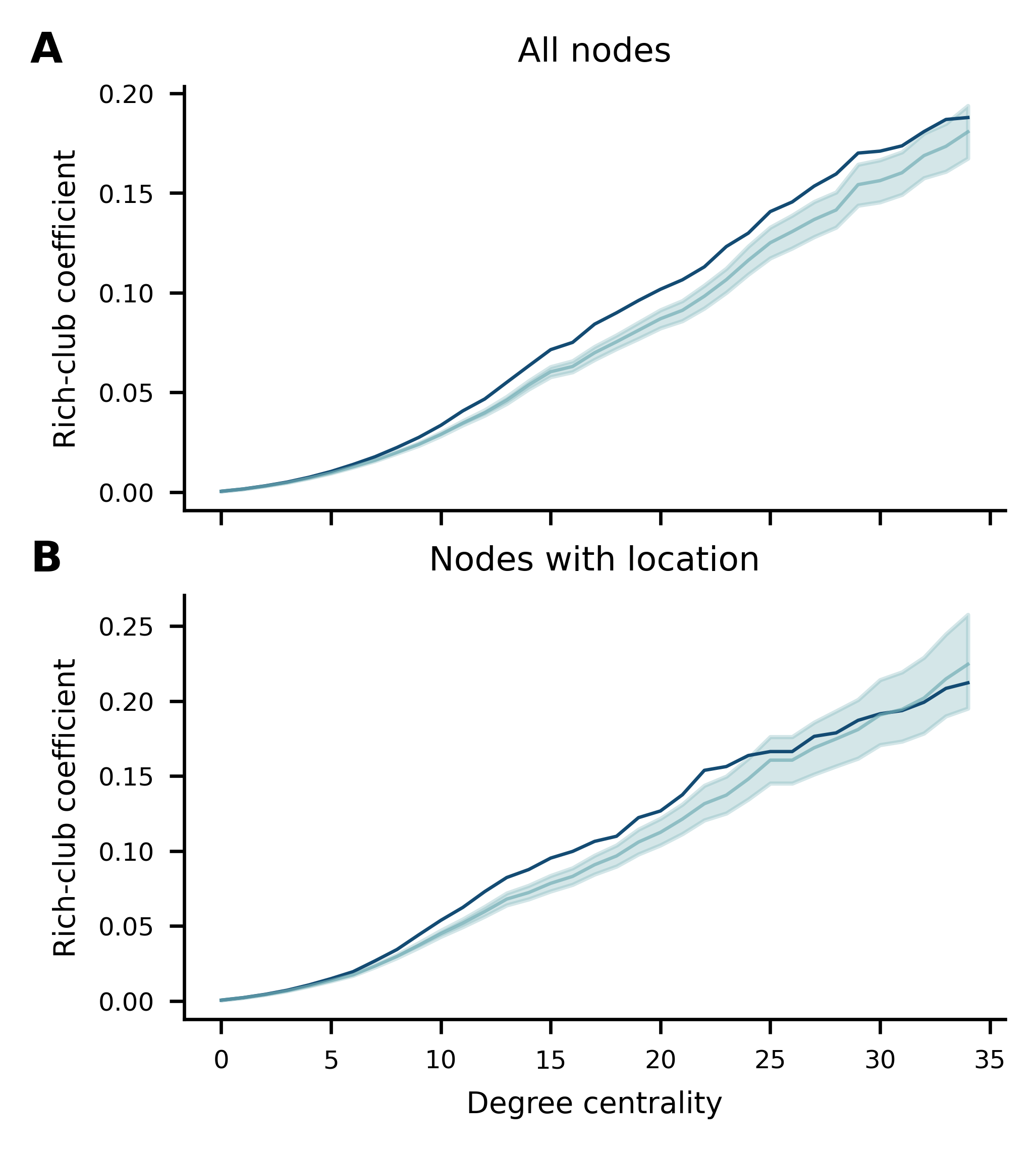

Supplement: Supplemental Information 4 — Rich-club coefficients (dark blue) at subsequent node degree centralities for (A) the whole network; and (B) the network composed of only nodes with location information. Rich-club coefficients from 500 randomised networks are shown in light blue, along with shaded 95\% confidence intervals. [file peerj-11-15270-s004.png]

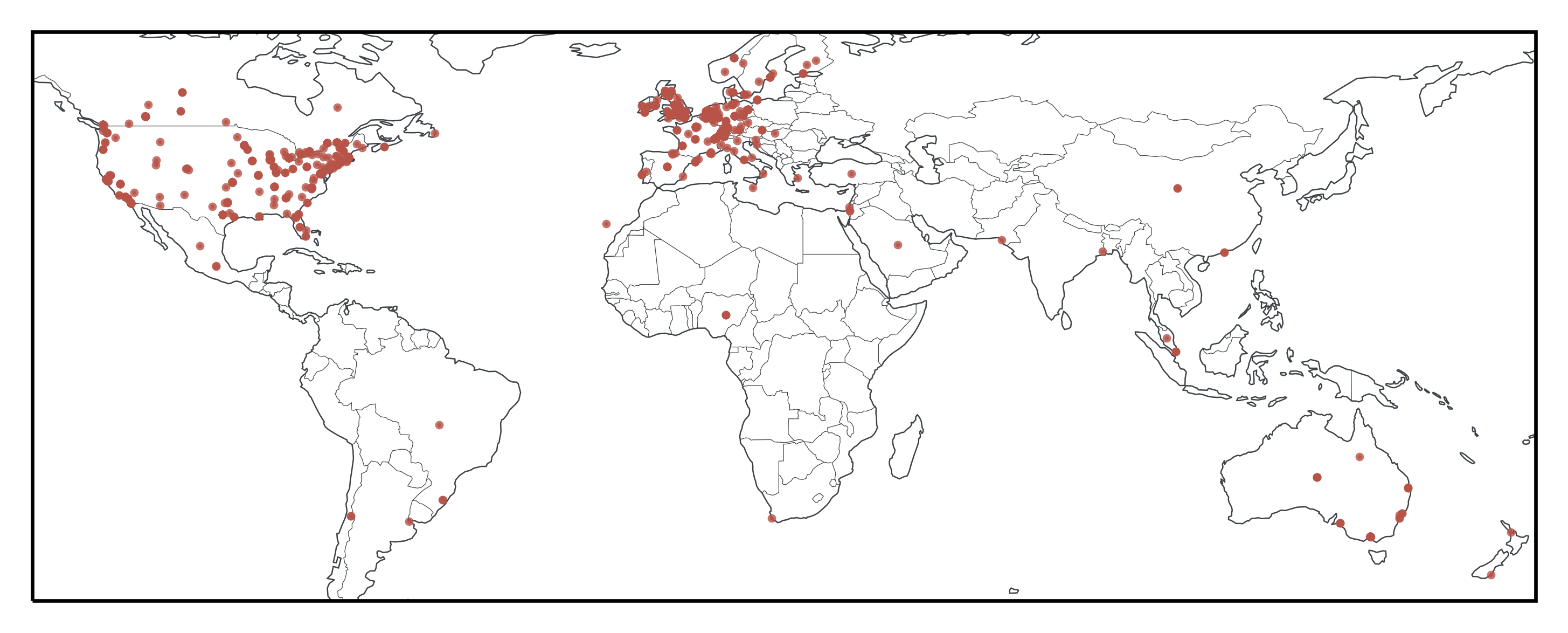

Supplement: Supplemental Information 5 [file peerj-11-15270-s005.png]

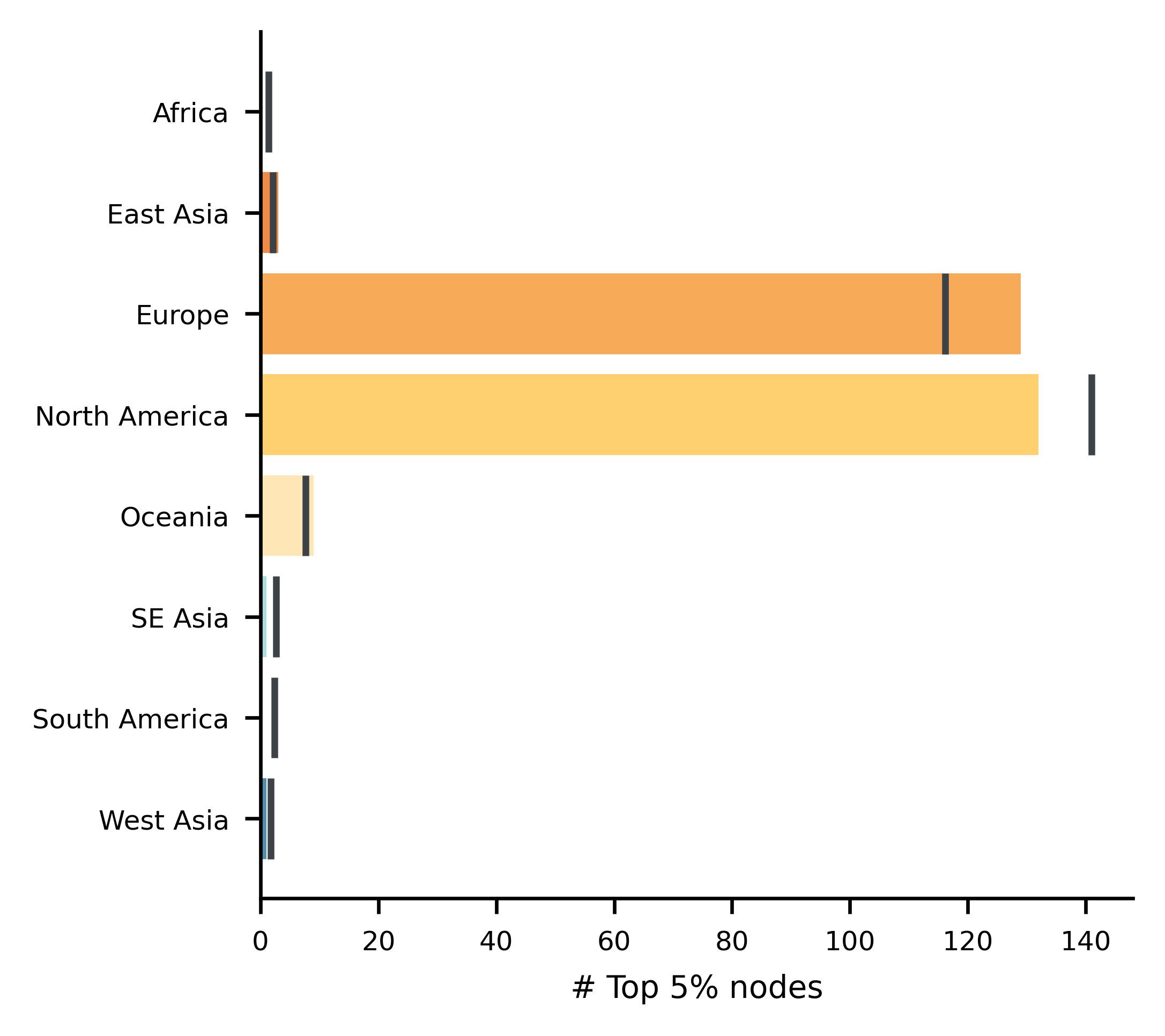

Supplement: Supplemental Information 6 — Number of top 5% important nodes per region. Expected numbers were important nodes to be distributed evenly across regions are shown with dark lines. [file peerj-11-15270-s006.png]
