# Supplementary material for: Analysing the effectiveness of Twitter as an equitable community communication tool for international conferences
Source: PeerJ. 2023 May 8;11:e15270. doi: 10.7717/peerj.15270 (PMC10174057; doi:10.7717/peerj.15270)
Supplement: Supplemental Information 7 [file peerj-11-15270-s007.pdf]

Region	Country	Region	Country	Region	Country
Africa	Ethiopia	Europe	Ireland	SE Asia	Bangladesh
	Ghana		Italy		India
	Kenya		Jersey		Indonesia
	Nigeria		Kosovo		Malaysia
	S Africa		Latvia		Nepal
	Tanzania		Lithuania		Pakistan
	Zimbabwe		Luxembourg		Philippines
	Algeria		Malta		Singapore
	Egypt		Netherlands		Sri Lanka
	Morocco		Norway		Thailand
East Asia	Cambodia		Poland		Vietnam
	China		Portugal	S America	Argentina
	Hong Kong		Romania		Brazil
	Japan		Russia		Chile
	Macao		San Marino		Colombia
	S Korea		Serbia		Dom. Republic
	Taiwan		Slovakia		Ecuador
Europe	Austria		Slovenia		Peru
	Belgium		Spain		Uruguay
	Bosnia		Sweden		Venezuela
	Bulgaria		Switzerland	West Asia	Iran
	Croatia		UK		Iraq
	Cyprus		Ukraine		Israel
	Czechia	N America	Canada		Kazakhstan
	Denmark		Costa Rica		Kuwait
	Estonia		Cuba		Lebanon
	Finland		Mexico		Oman
	France		Puerto Rico		Qatar
	Germany		USA		Saudi Arabia
	Greece	Oceania	Australia		Turkey
	Hungary		New Zealand		UAE
	Iceland				

Table S1: Region assignment for each country.
