# Supplementary material for: Analysing the effectiveness of Twitter as an equitable community communication tool for international conferences
Source: PeerJ. 2023 May 8;11:e15270. doi: 10.7717/peerj.15270 (PMC10174057; doi:10.7717/peerj.15270)
Supplement: Supplemental Information 8 [file peerj-11-15270-s008.pdf]

Journal	Publications	With location	Excluded
eNeuro	1581	1436	9.2%
Eur J Neurosci	4019	3930	2.2%
Front Hum Neurosci	6390	6318	1.1%
Front Neurosci	7042	7019	0.3%
Hum Brain Mapp	3692	3638	1.5%
J Neurosci	14796	13176	10.9%
Magn Reson Med	5196	5162	0.7%
NeuroImage	10970	10804	1.5%

Table S2: Publications with location information from each journal.
