# Supplementary material for: Analysing the effectiveness of Twitter as an equitable community communication tool for international conferences
Source: PeerJ. 2023 May 8;11:e15270. doi: 10.7717/peerj.15270 (PMC10174057; doi:10.7717/peerj.15270)
Supplement: Supplemental Information 9 [file peerj-11-15270-s009.pdf]

Country	Users
Bosnia and Herzegovina	2
Cambodia	1
Costa Rica	1
Ecuador	1
Ghana	1
Jersey	1
Latvia	1
Macao	1
Nepal	2
Peru	1
Puerto Rico	9

Table S3: Countries with Twitter users but no publications.
